# Supplementary material for: The Antineoplastic Effect of Carboplatin Is Potentiated by Combination with Pitavastatin or Metformin in a Chemoresistant High-Grade Serous Carcinoma Cell Line
Source: Int J Mol Sci. 2022 Dec 21;24(1):97. doi: 10.3390/ijms24010097 (PMC9820586; doi:10.3390/ijms24010097)
Supplement: Supplementary file 1 [file ijms-24-00097-s001.zip › ijms-2120665-supplementary.pdf]

Supplementary Material

Table S1. Cellular viability (%) of OVCAR8 and OVCAR8 PTX R P cells, after exposure to a fixed doses ratio that corresponds to 0.25, 0.5, 1, 2 and 4 times the individual IC_{50} values of each drug for 48 h. The combined treatment was co-administered at the same time. All assays were done in triplicate in at least three independent experiments. SD, standard deviation.

Treatment	Concentration	OVCAR8 Cellular viability (%)		OVCAR8 PTX R P Cellular viability (%)	
		**Media**	**SD**	**Media**	**SD**
**Carboplatin (μM)**	0.25 x IC50	92.531	0.672	87.570	1.918
	0.5 x IC50	78.349	8.436	75.397	3.802
	IC50	60.702	5.190	57.172	4.484
	2 x IC50	9.962	2.159	24.033	8.183
	4 x IC50	3.178	1.576	3.973	1.422
**Pitavastatin (μM)**	0.25 x IC50	60.608	3.877	60.785	7.665
	0.5 x IC50	52.738	3.251	49.751	2.207
	IC50	46.854	2.547	44.716	1.406
	2 x IC50	35.398	3.108	28.515	4.045
	4 x IC50	29.124	6.221	28.178	3.446
**Carboplatin (μM) + Pitavastatin (μM)**	0.25 x IC50	60.349	2.856	56.075	10.193
	0.5 x IC50	48.552	1.908	40.283	6.228
	IC50	31.048	1.237	25.796	3.497
	2 x IC50	19.453	4.417	12.832	8.774
	4 x IC50	5.576	0.735	4.173	2.130
**Metformin (mM)**	0.25 x IC50	73.629	4.867	75.521	1.641
	0.5 x IC50	60.764	1.710	60.300	1.300
	IC50	46.377	4.049	45.069	0.889
	2 x IC50	19.461	2.920	12.592	1.286
	4 x IC50	15.469	3.467	7.795	1.018
**Carboplatin (μM) + Metformin (mM)**	0.25 x IC50	70.624	3.533	73.912	1.977
	0.5 x IC50	54.726	1.645	55.091	4.483
	IC50	8.641	1.962	9.897	3.787
	2 x IC50	7.105	1.963	8.138	1.219
	4 x IC50	4.060	0.503	3.435	1.049
**Ivermectin (μM)**	0.25 x IC50	76.543	1.978	81.214	2.466
	0.5 x IC50	68.485	0.746	64.803	2.168
	IC50	42.511	1.788	44.580	3.147
	2 x IC50	0.514	0.050	1.210	1.256
	4 x IC50	0.379	0.299	0.494	0.183
**Carboplatin (μM) + Ivermectin (μM)**	0.25 x IC50	91.320	7.718	95.832	3.646
	0.5 x IC50	74.018	11.931	73.639	9.023
	IC50	32.828	3.453	25.286	3.508
	2 x IC50	0.290	0.214	1.644	1.348
	4 x IC50	0.020	0.451	0.593	0.905
**Itraconazole (μM)**	0.25 x IC50	94.332	7.175	90.418	14.825
	0.5 x IC50	95.498	3.874	97.015	19.576

	IC50	95.141	6.063	94.016	16.247
	2 x IC50	98.250	2.400	91.571	13.226
	4 x IC50	98.398	7.288	89.624	7.916
**Carboplatin (μM) + Itraconazole (μM)**	0.25 x IC50	89.970	1.158	90.445	10.179
	0.5 x IC50	80.492	3.557	76.639	6.622
	IC50	63.513	3.335	56.847	8.386
	2 x IC50	44.603	4.434	28.241	8.300
	4 x IC50	4.998	3.142	3.299	1.794
**Alendronate (μM)**	0.25 x IC50	99.951	1.327	98.798	3.424
	0.5 x IC50	92.024	2.157	85.644	0.167
	IC50	44.307	4.276	46.798	3.093
	2 x IC50	37.529	4.275	41.214	5.101
	4 x IC50	31.770	5.646	40.422	5.688
**Paclitaxel (nM) + Alendronate (μM)**	0.25 x IC50	92.829	5.804	92.727	6.105
	0.5 x IC50	80.026	9.039	75.374	11.574
	IC50	29.772	1.781	30.868	3.128
	2 x IC50	18.664	3.727	16.565	3.230
	4 x IC50	6.752	0.061	6.396	1.081

Table S2. Cellular viability (%) of HOSE6.3 cells, after exposure to a fixed doses ratio that corresponds to 0.25, 0.5, 1, 2 and 4 times the individual IC_{50} values of each drug for 48 h. The combined treatment was co-administered at the same time. All assays were done in triplicate in at least three independent experiments. SD, standard deviation.

Treatment	Concentration	HOSE6.3	
		Cellular viability (%)	
		Media	SD
**Carboplatin (µM)**	0.25 × IC50	88.270	5.412
	0.5 × IC50	80.640	1.159
	IC50	72.731	4.688
	2 × IC50	73.328	1.388
	4 × IC50	51.060	2.693
**Pitavastatin (µM)**	0.25 × IC50	81.849	7.680
	0.5 × IC50	76.512	7.815
	IC50	68.461	3.472
	2 × IC50	65.889	5.045
	4 × IC50	61.442	2.761
**Carboplatin (µM) + Pitavastatin (µM)**	0.25 × IC50	85.461	3.279
	0.5 × IC50	85.366	2.539
	IC50	78.648	2.658
	2 × IC50	70.445	0.900
	4 × IC50	53.617	2.412
**Metformin (mM)**	0.25 × IC50	96.286	4.708
	0.5 × IC50	97.225	1.002
	IC50	95.033	1.429
	2 × IC50	96.033	1.778
	4 × IC50	92.366	2.639
**Carboplatin (µM) + Metformin (mM)**	0.25 × IC50	99.735	1.395
	0.5 × IC50	94.038	1.253
	IC50	86.581	1.751
	2 × IC50	73.321	2.244
	4 × IC50	60.842	0.441
**Ivermectin (µM)**	0.25 × IC50	100.990	2.144
	0.5 × IC50	99.694	3.725
	IC50	53.462	2.873
	2 × IC50	0.539	1.365
	4 × IC50	0.000	0.544
**Carboplatin (µM) + Ivermectin (µM)**	0.25 × IC50	99.024	1.122
	0.5 × IC50	99.061	1.886
	IC50	47.689	3.598
	2 × IC50	2.2495	0.514
	4 × IC50	1.942	0.937
**Itraconazole (µM)**	0.25 × IC50	86.353	3.252
	0.5 × IC50	84.356	1.857
	IC50	84.735	3.865
	2 × IC50	79.729	1.179
	4 × IC50	77.370	3.174

**Carboplatin (µM) + Itraconazole (µM)**	0.25 × IC50	99.610	1.337
	0.5 × IC50	92.847	1.446
	IC50	87.060	1.599
	2 × IC50	74.815	3.392
	4 × IC50	62.169	0.574
**Alendronate (µM)**	0.25 × IC50	101.792	6.109
	0.5 × IC50	101.959	6.966
	IC50	92.147	2.125
	2 × IC50	89.188	5.221
	4 × IC50	84.305	5.970
**Carboplatin (µM) + Alendronate (µM)**	0.25 × IC50	99.274	0.455
	0.5 × IC50	96.901	0.646
	IC50	94.532	3.028
	2 × IC50	92.908	2.486
	4 × IC50	76.021	3.396
